# Supplementary material for: Performance boost for bismuth telluride thermoelectric generator via barrier layer based on low Young’s modulus and particle sliding
Source: Nat Commun. 2023 Dec 6;14:8085. doi: 10.1038/s41467-023-43879-8 (PMC10700503; doi:10.1038/s41467-023-43879-8)
Supplement: Supplementary file 1 — Supplementary Information [file 41467_2023_43879_MOESM1_ESM.pdf]

Supporting Information

Performance Boost for Bismuth Telluride Thermoelectric Generator via Barrier Layer Based on Low Young's Modulus and Particle Sliding

Yuxin Sun[1]†, Fengkai Guo[1]†*, Yan Feng[2], Chun Li[3], Yongchun Zou[4], Jinxuan Cheng[5], Xingyan Dong[1], Hao Wu[1], Qian Zhang[5], Weishu Liu[6], Zihang Liu[3], Wei Cai[1], Zhifeng Ren[7]*, Jiehe Sui[1]*

1 National Key Laboratory for Precision Hot Processing of Metals, Harbin Institute of Technology; Harbin, 150001, China
2 State Key Laboratory of Solidification Processing, Northwestern Polytechnical University; Xi'an, 710072, China
3 State Key Laboratory of Advanced Welding and Joining, Harbin Institute of Technology; Harbin, 150001, China
4 Center of Analysis Measurement and Computing, Harbin Institute of Technology; Harbin, 150001, China
5 School of Materials Science and Engineering, and Institute of Materials Genome & Big Data; Harbin Institute of Technology, Shenzhen, 518055, China
6 Department of Materials Science and Engineering, Southern University of Science and Technology, Shenzhen, 518055, China
7 Department of Physics and Texas Center for Superconductivity at the University of Houston (TcSUH), University of Houston, Houston, TX 77204, USA

†These authors contributed equally to this work.
*To whom correspondence should be addressed. E-mail: fkguo@hit.edu.cn, zren@uh.edu, suijiehe@hit.edu.cn

Supplementary Note 1: Young's modulus and Poisson's ratio calculations

Poisson's ratio (μ) is calculated using:

$$\mu = \frac{1}{2}\frac{(v_l/v_t)^2-2}{(v_l/v_t)^2-1},$$

where v_l is longitudinal wave velocity and v_t is transverse wave velocity.

Young's modulus (E) is calculated using:

$$E = \rho v_t^2 \frac{3v_l^2-4v_t^2}{v_l^2-v_t^2},$$

where ρ is density.

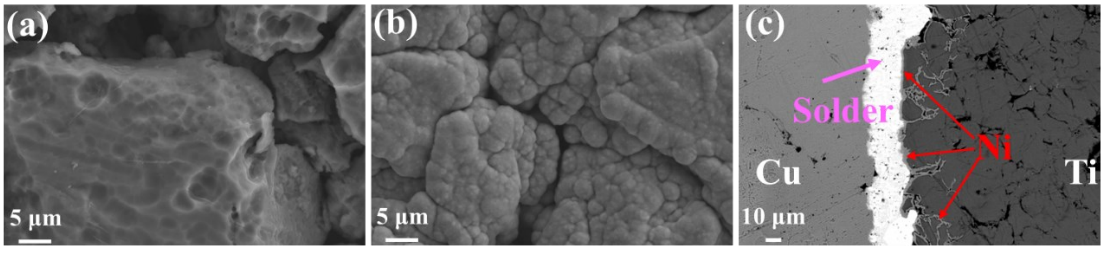

Supplementary Fig. 1. Morphology of electroplated Ni on Ti Surface. Secondary electron image of Ti layer surface following (a) corrosion and (b) electroplating. (c) Backscattered electron (BSE) image of the Cu/solder/Ni/Ti interface showing excellent connection quality.

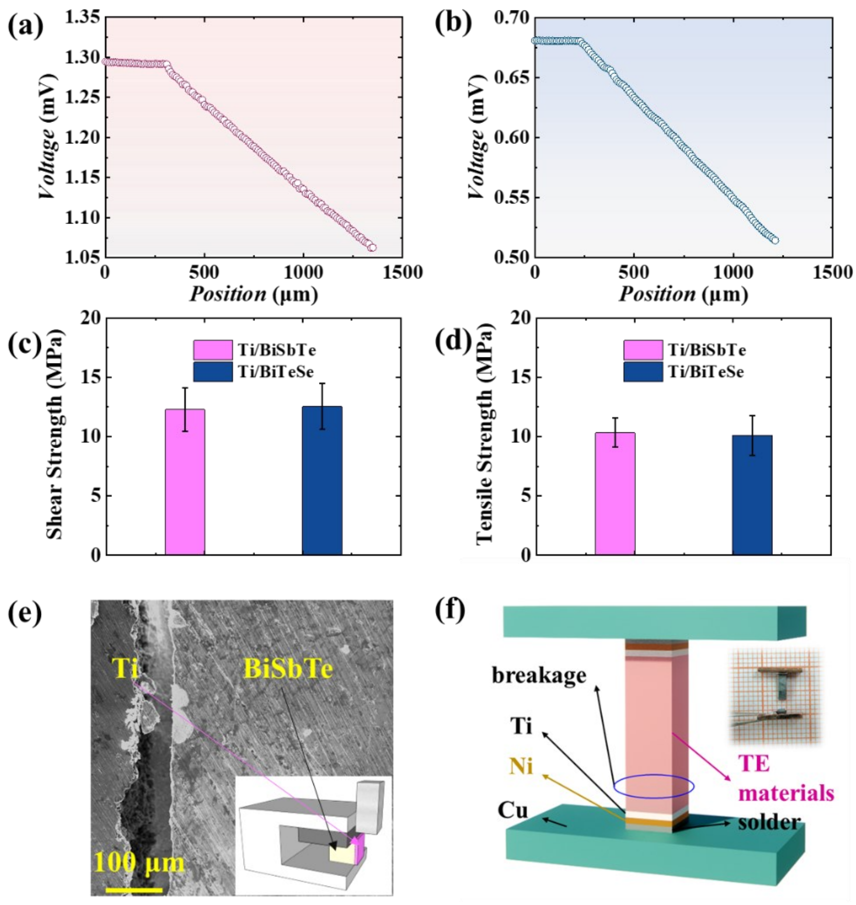

Supplementary Fig. 2. Functional characteristics of Ti/BiTe joints. Contact resistivity curves of as-sintered (a) Ti/BiSbTe and (b) Ti/BiTeSe joints. (c) Shear strength and (d) tensile strength of Ti/BiSbTe and Ti/BiTeSe joints at room temperature. (e) Scanning electron microscopy (SEM) image of the Ti/BiSbTe joint after shearing. Inset: schematic illustration of the shear test. (f) Schematic diagram of the tensile test. Inset: Photograph of the stretched sample. Since the fracture occurs inside the TE material, the tensile strength of the joint should be higher than the measured value.

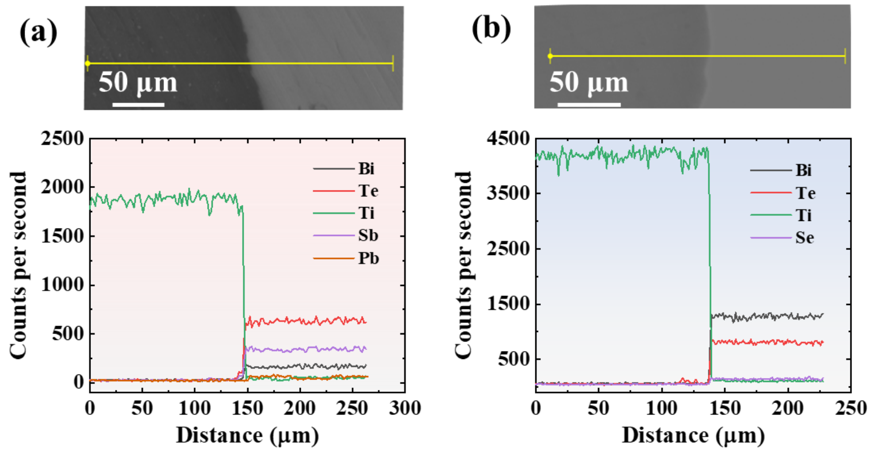

Supplementary Fig. 3. SEM and Energy dispersive spectroscopy (EDS) line scanning results of Ti/BiTe joints. BSE images and compositional line profiles of the (a) Ti/BiSbTe and (b) Ti/BiTeSe joints.

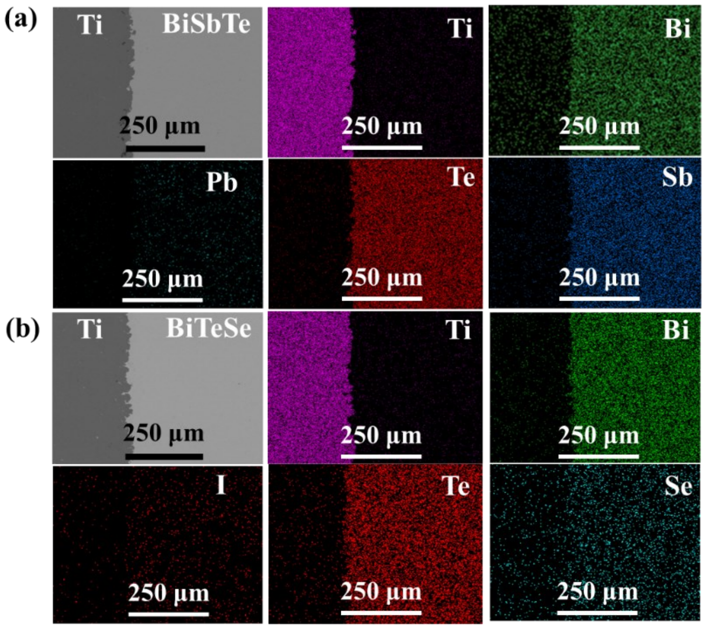

Supplementary Fig. 4. EDS mapping of Ti/BiTe joints. (a) Ti/BiSbTe joint, (b) Ti/BiTeSe joint.

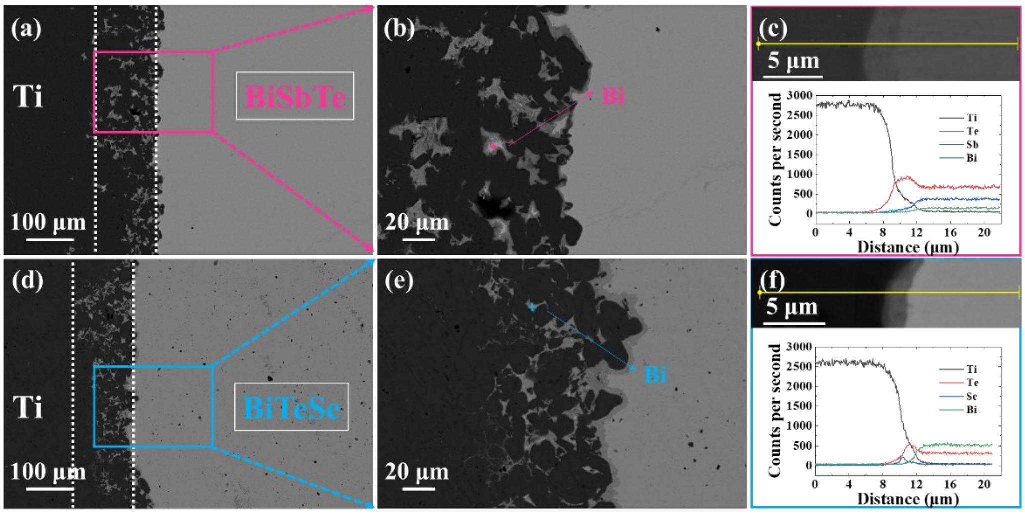

Supplementary Fig. 5. BSE images of Ti/BiTe joints sintered at 773 K for 30 min. BSE images of (a) Ti/BiSbTe and (d) Ti/BiTeSe. (b) and (e) are enlarged views of the selected interface areas in (a) and (d), respectively. (c) and (f) are elemental composition profiles along the yellow lines for the Ti/BiSbTe and Ti/BiTeSe joints, respectively.

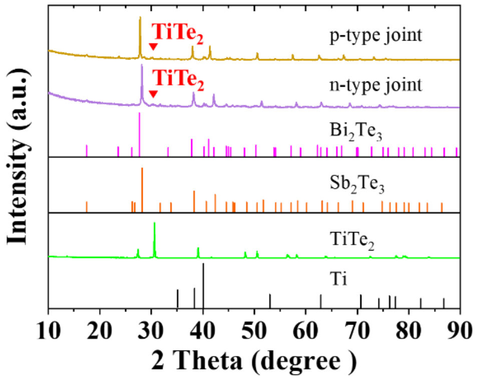

Supplementary Fig. 6. X-ray diffraction (XRD) patterns of cross-sections of the Ti/BiSbTe and Ti/BiTeSe joints.

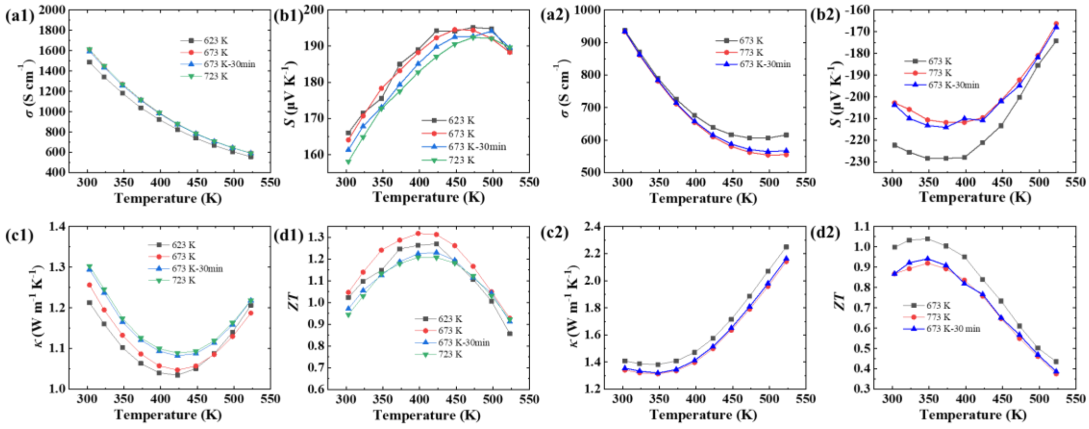

Supplementary Fig. 7. Thermoelectric properties for BiTe under different sintering conditions. (a1) - (d1) Thermoelectric properties of $Bi_{0.399}Sb_{1.596}Pb_{0.005}Te_3$ sintered at 623 K, 673 K, 723 K for 5 min, and 673 K for 30 min. (a2) - (d2) Thermoelectric properties of extruded n-type BiTe re-sintered at 673 K, 723 K for 5 min and 673 K for 30 min.

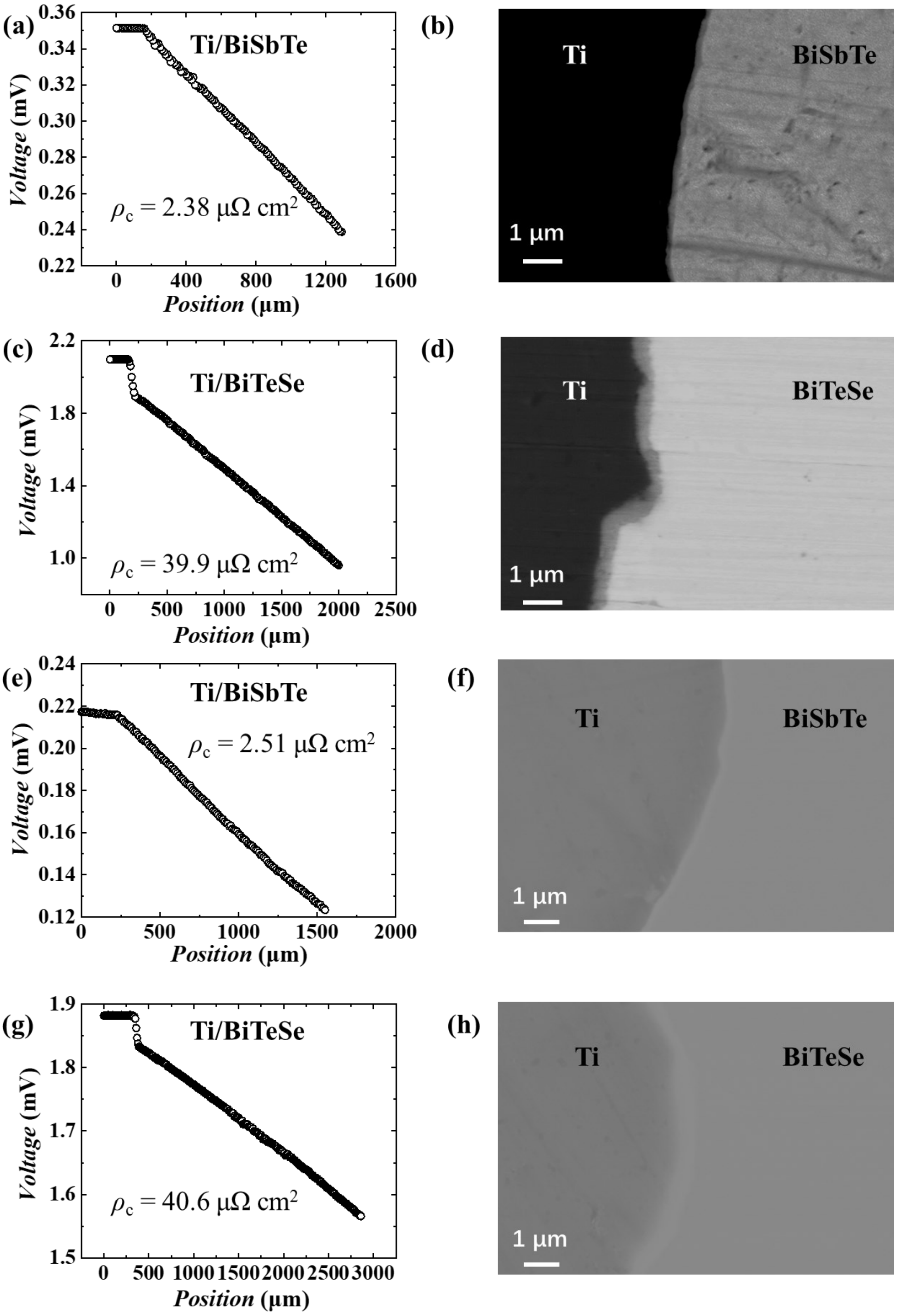

Supplementary Fig. 8. Contact resistivity curves and BSE images of Ti/BiTe joints by extending sintering time or increase sintering temperature. Contact resistivity curves (a) (c) and BSE images (b) (d) of Ti/BiSbTe and Ti/BiTeSe joints sintered at 673 K for 30 min. Contact resistivity curves (e) (f) and BSE images (g) (h) of Ti/BiSbTe and Ti/BiTeSe joints sintered at 723 K for 5 min.

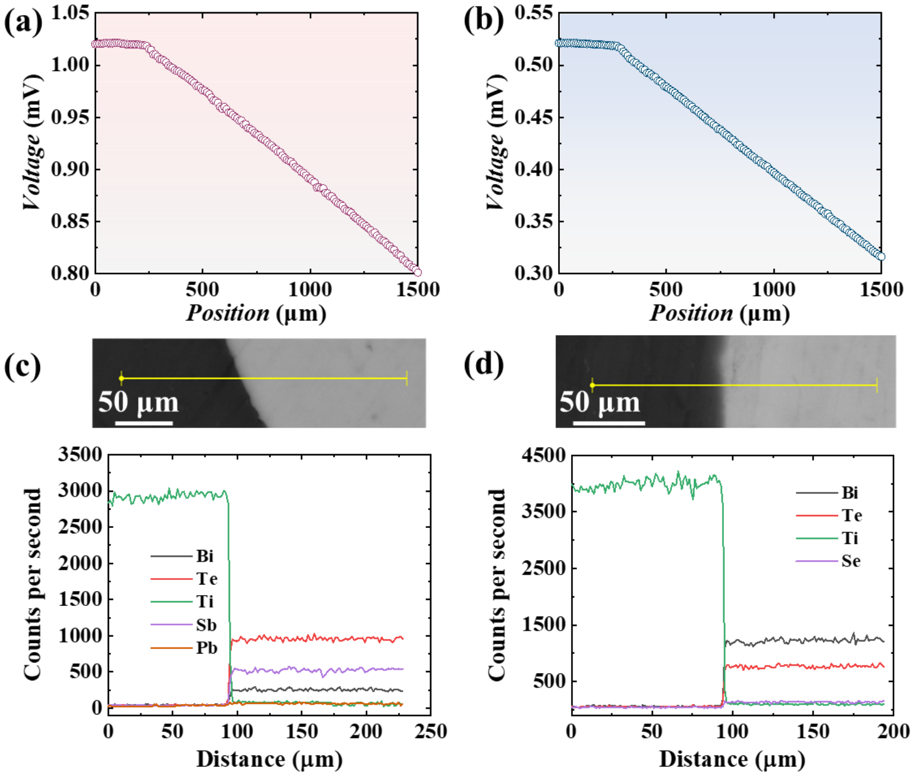

Supplementary Fig. 9. Contact resistivity curves and SEM results of Ti/BiTe joints after aging at 523 K for 45 days. Contact resistivity curves for (a) Ti/BiSbTe and (b) Ti/BiTeSe joints. BSE images and corresponding compositional line profiles of (c) Ti/BiSbTe and (d) Ti/BiTeSe joints.

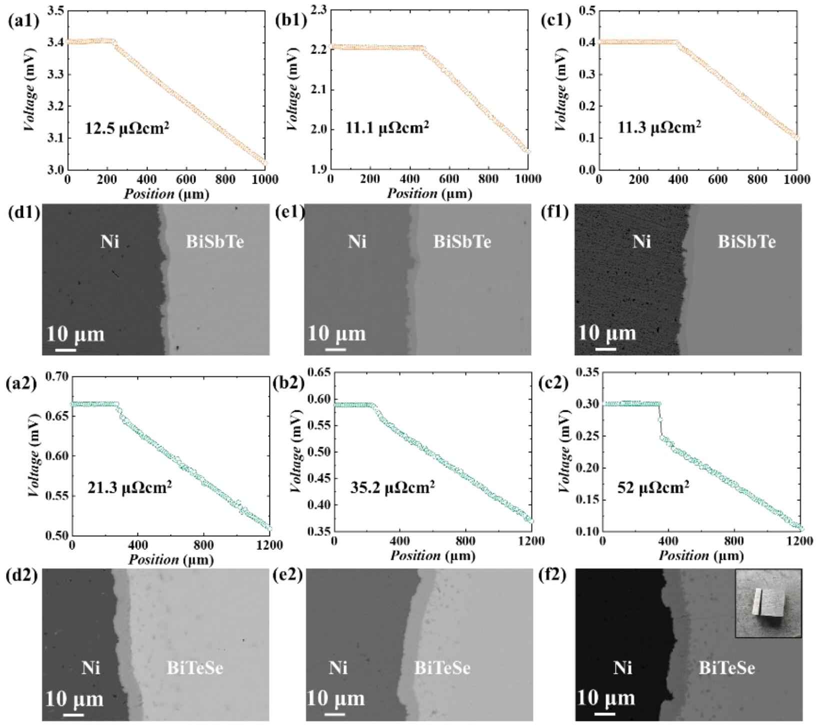

Supplementary Fig. 10. Contact resistivity curves and BSE images of Ni/BiTe joints. (a-c) ρ_c values and (d-f) corresponding BSE images of (1) Ni/BiSbTe and (2) Ni/BiTeSe joints after aging for 15, 30, or 45 days, respectively. The inset to f2 is a photograph showing sample

fracture during the grinding process.

Supplementary Table 1. The tensile strength and shear strength of Ni/BiTe joints at room temperature.

	Tensile strength[1]	Shear strength
Ni/p-type BiTe	~30 MPa	~14 MPa
Ni/n-type BiTe	~16 MPa	~16 MPa

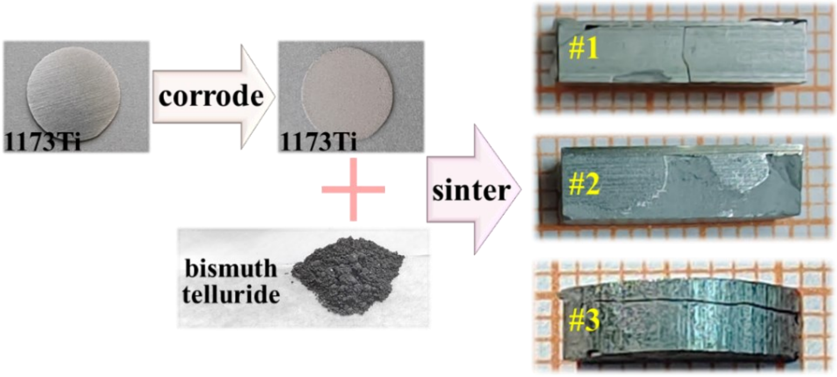

Supplementary Fig. 11. Sintering process for the 1173Ti/bismuth telluride joint and resulting products. #1-#3 represent three different samples obtained.

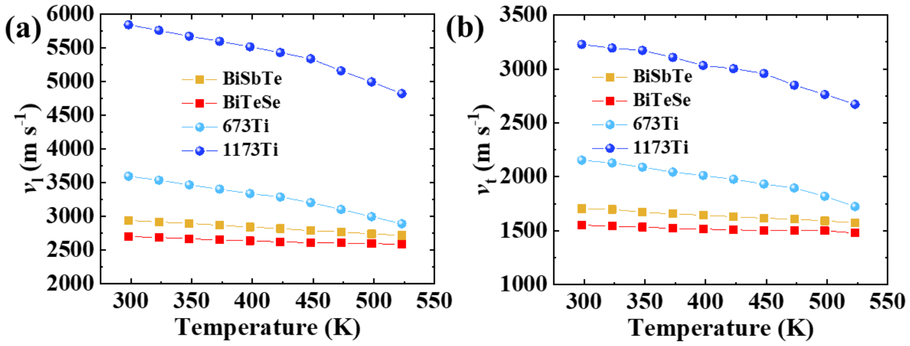

Supplementary Fig. 12. Temperature-dependent sound velocities of BiSbTe, BiTeSe, 673Ti, and 1173Ti. (a) Longitudinal velocities v_l, (b) transverse velocities v_t.

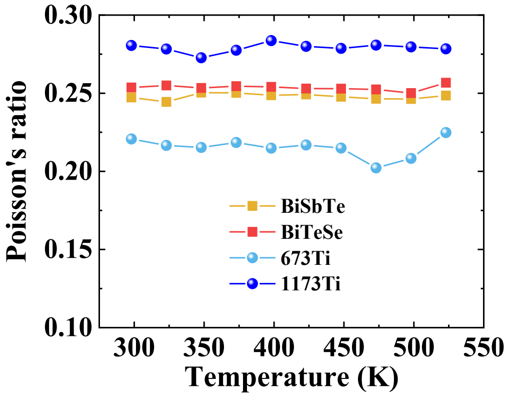

Supplementary Fig. 13. Temperature-dependent Poisson's ratio of BiSbTe, BiTeSe, 673Ti, and 1173Ti calculated using ν_{t} and ν_{l}.

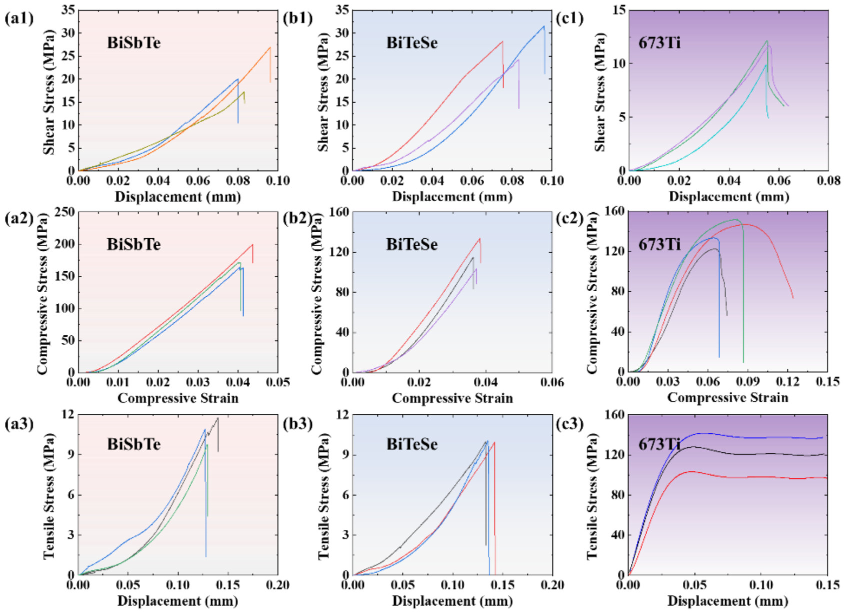

Supplementary Fig. 14. Mechanical properties of BiSbTe, BiTeSe, and 673Ti. (a1-c1) Shear, (a2-c2) compressive, and (a3-c3) tensile stress curves. Different colored curves represent different samples, and at least three samples were measured for each test.

Supplementary Table 2. Shear stress, compressive stress, tensile strength, and density values of BiSbTe, BiTeSe, and 673Ti at room temperature (RT).

Material	Shear stress (MPa)	Compressive stress (MPa)	Tensile strength (MPa)	Density (g/cm^3)
BiSbTe	21.36	178.4	10.8	6.6
BiTeSe	28	117.57	10.1	7.7
673Ti	11.2	138	127.3	3.4

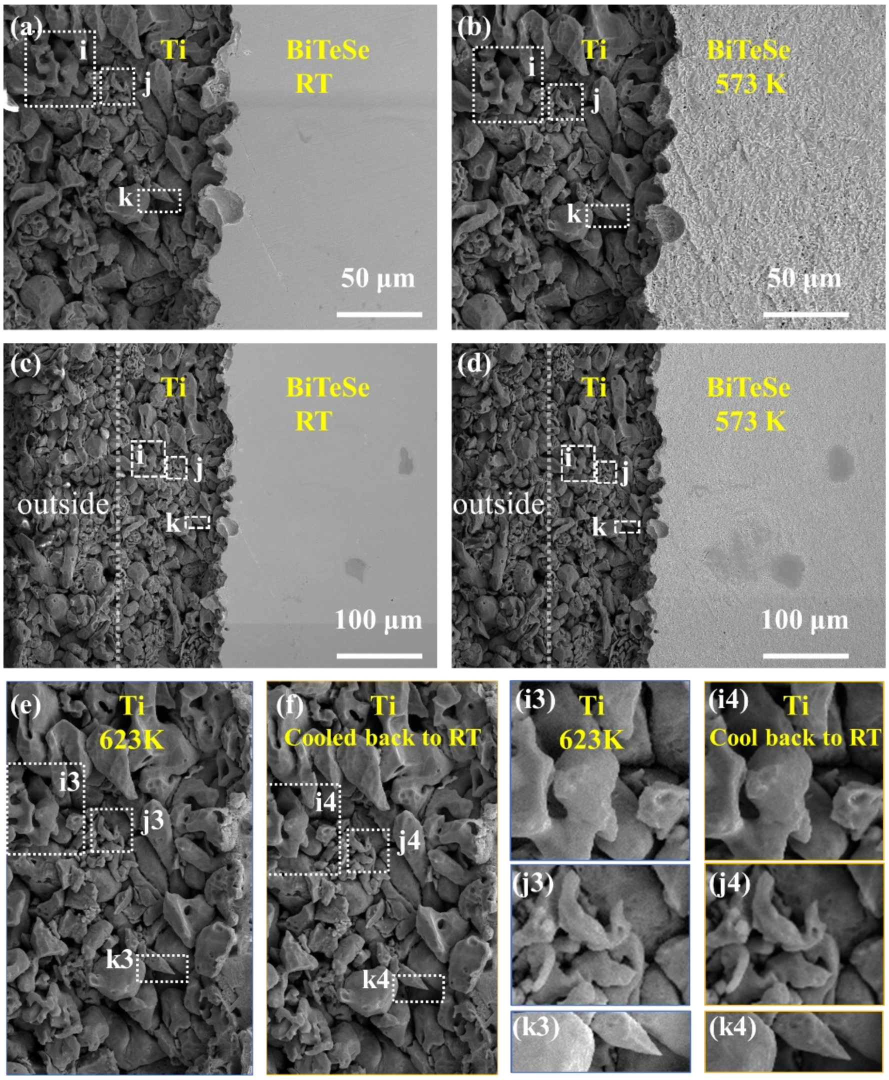

Supplementary Fig. 15. SEM images of the Ti/BiTeSe joint at different states. (a) and (c) RT, (b) and (d) 573 K. (a) and (b) are magnified views of (c) and (d), respectively. SEM images of the Ti/BiTeSe joint (e) at 623 K and (f) after cooling back to RT. (i3-k3) and (i4-k4) are magnified views of the corresponding marked regions in (e) and (f), respectively.

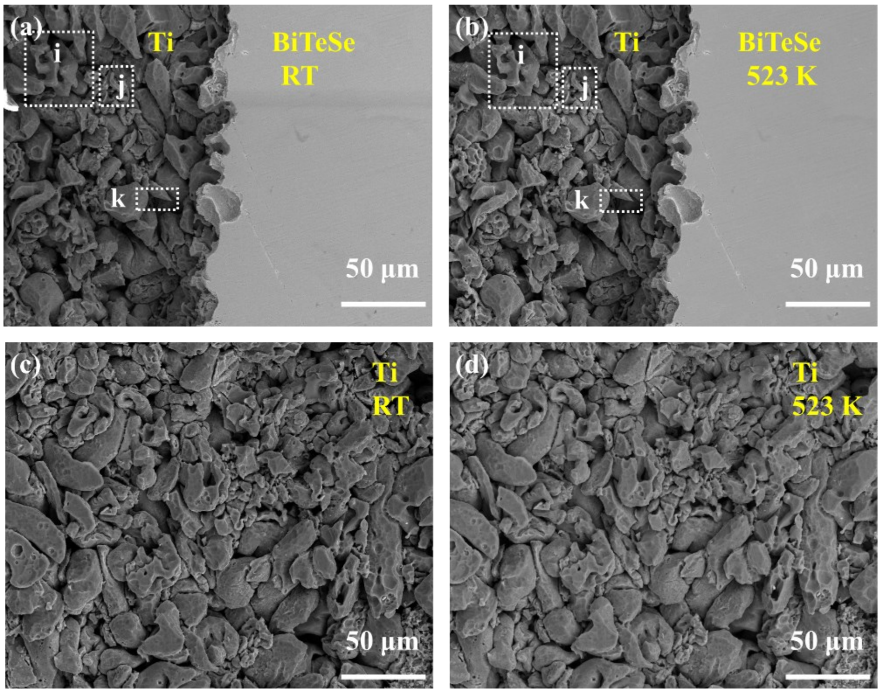

Supplementary Fig. 16. SEM images of the interface and Ti particles in the Ti/BiTeSe joint at different temperatures. (a) RT, (b) 523 K. SEM images of a new sample prepared to demonstrate once again that there is no particle sliding when the temperature is raised from (c) RT to (d) 523 K.

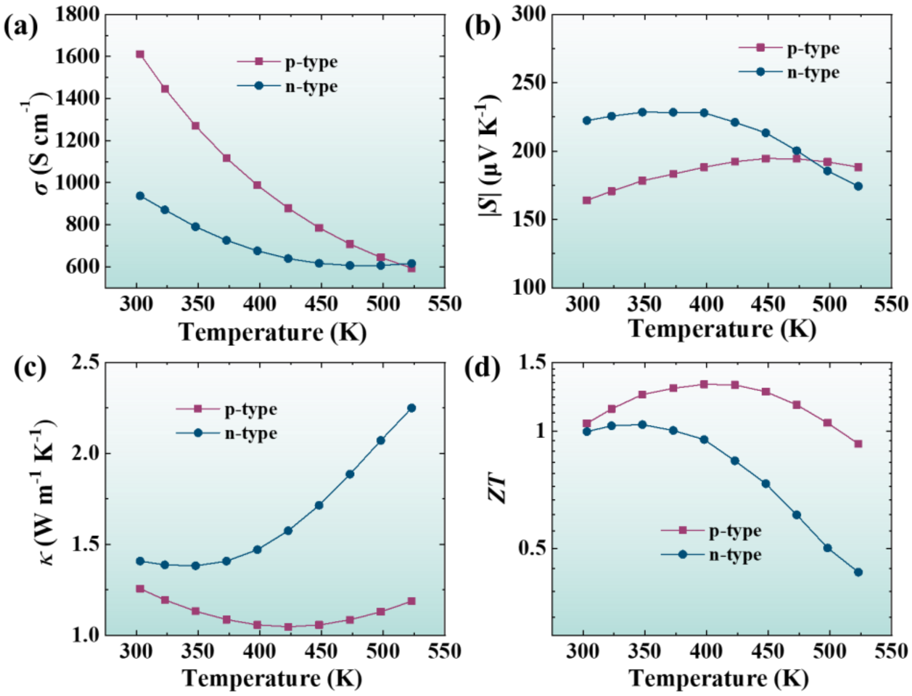

Supplementary Fig. 17. Temperature-dependent thermoelectric properties of $Bi_{0.399}Sb_{1.596}Pb_{0.005}Te_3$ and n-type commercial extruded bismuth telluride. (a) Electrical conductivity σ, (b) absolute value of Seebeck coefficient $|S|$, (c) thermal conductivity κ, and (d) ZT value.

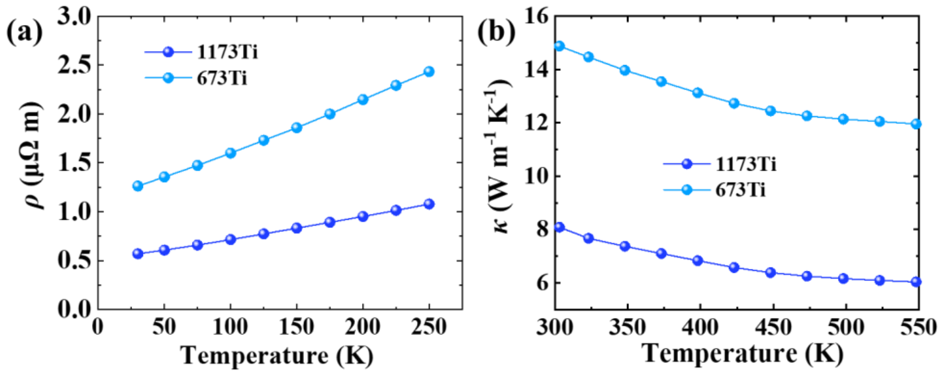

Supplementary Fig. 18. Temperature-dependent transport properties of 673Ti and 1173Ti. (a) Resistivity, (b) thermal conductivity.

References

1 W. Liu, H. Wang, L. Wang, X. Wang, G. Joshi, G. Chen and Z. Ren, Understanding of the contact of nanostructured thermoelectric n-type Bi2Te2.7Se0.3 legs for power generation applications, *J. Mater. Chem. A*, 2013, **1**, 13093.
